# Selection on a Neural Gene Regulator Sheds Light on Human Evolution

**DOI:** 10.1371/journal.pbio.0030417

**Published:** 2005-11-15

With humans and chimpanzees differing by just 1.2% at the DNA level, it's clear that our differences do not arise from gene variation alone. Thirty years ago, Mary-Claire King and Alan Wilson pointed to our extensive protein similarities as evidence that those investigating the genetic basis of human origins should focus on the regulators of gene expression rather than on the genes themselves.

DNA sequences that regulate gene expression, called *cis*-regulatory elements, occur on the same DNA molecule as the regulated gene. By recruiting proteins that initiate or block transcription, *cis*-regulatory elements influence the rate at which genes are transcribed, in which cells, and under what conditions. But since sequence inspection doesn't reveal whether regulatory sequence changes are functional or neutral, finding evidence for human-specific changes and positive selection in these sequences is far harder than finding similar evidence in protein sequences or in gene sets. Now, Matthew Rockman, Gregory Wray, and their colleagues provide further support for King and Wilson's predictions by showing that positive selection altered the *cis*-regulation of a gene expressed in the human brain.

To find evidence of regulatory changes underlying uniquely human traits, Rockman et al. examined the regulatory evolution of *prodynorphin*, a gene expressed in multiple brain and endocrine cell types. The protein encoded by *prodynorphin* is a precursor molecule for a suite of neuropeptides that bind to opiate receptors and affect perception, pain sensation, emotion, and learning. In humans, *prodynorphin*'s promoter contains what's called a 68 base-pair tandem repeat polymorphism—individuals can have up to four copies of the 68 DNA base-pair element, which occur side by side. The polymorphism, which affects how many transcripts of the gene are produced, has been tentatively linked to schizophrenia, cocaine addiction, and epilepsy.

To explore how this functional variation evolved, Rockman et al. first sequenced and analyzed *prodynorphin* regulatory DNA from 74 human chromosomes and 32 nonhuman primate chromosomes (chimp, bonobo, gorilla, orangutan, baboon, and two macaque species). The duplication leading to tandem repeats appears unique to humans, since all the monkeys and other great apes carry only one copy of the 68 base-pair element. Further distinguishing humans from the last common ancestor of humans and chimps, the human copies also carry five mutations, or substitutions, far more than would be expected if the mutations were neutral (that is, had no effect on fitness). Three nearby polymorphisms also occurred at a higher-than-expected frequency in humans, a sign that selection acted on the linked neighboring sequences. The protein-coding sequence of *prodynorphin*, on the other hand, appears to have undergone negative selection, discarding harmful mutations that would disrupt its function. [Fig pbio-0030417-g001]

**Figure pbio-0030417-g001:**
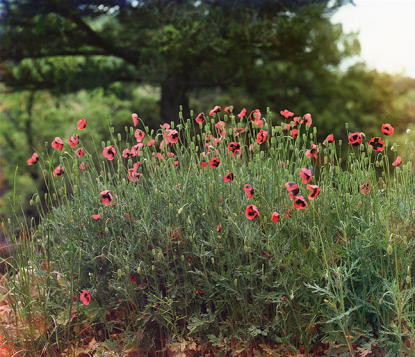
Positive natural selection altered the regulation of the human prodynorphin gene, which produces a product that interacts with the same receptors that opium targets

To determine the functional effects of the human substitution, the authors attached a bioluminescent enzyme to human and chimp *prodynorphin* *cis*-regulatory DNA, and introduced the modified DNA into human cell lines so they could measure transcription levels. Only the human 68 base-pair element significantly increased transcription of *prodynorphin*, and this increase was seen only in brain cells.

Because different numbers of repeats are associated with different effects, such as protection against cocaine addiction and neurological disease, Rockman et al. searched for signs of recent selection. If selection had occurred, divergence among populations should be increased and variation within populations reduced, relative to the neutral case. And that's what the authors found. The frequency of three-repeat versions of the 68 base-pair element was less than 10% in Chinese and Papua New Guinean populations and over 60% in Italy and Ethiopia. And variation at genome markers called microsatellites was significantly low overall across most of the populations studied (which also included India). Because microsatellites typically undergo high mutation rates, if a microsatellite is linked to an element under positive selection, it should show reduced variation.

The observed pattern of variations within and among human populations, the authors argue, suggests that recent selection has favored different versions of *prodynorphin*-regulatory elements in different regions of the world. These results support the longstanding notion that changes in gene regulation had major impacts on the evolution of novel traits, and may well hold the key to that eternal question, what makes us human? —*Liza Gross*

